# Longitudinal Associations of Toothbrushing With Obesity and Hyperglycemia

**DOI:** 10.2188/jea.JE20190165

**Published:** 2020-12-05

**Authors:** Michiko Furuta, Kenji Takeuchi, Toru Takeshita, Akihiko Tanaka, Shino Suma, Takashi Shinagawa, Yoshihiro Shimazaki, Yoshihisa Yamashita

**Affiliations:** 1 Section of Preventive and Public Health Dentistry, Division of Oral Health, Growth and Development, Faculty of Dental Science, Kyushu University, Fukuoka, Japan; 2 Department of Preventive Medicine, Nagoya University Graduate School of Medicine, Nagoya, Japan; 3 OBT Research Center, Faculty of Dental Science, Kyushu University, Fukuoka, Japan; 4 Department of Dentistry and Oral Surgery, Heisei Yokohama Hospital, Yokohama, Japan; 5 Department of Preventive Dentistry and Dental Public Health, School of Dentistry, Aichi-Gakuin University, Nagoya, Japan

**Keywords:** toothbrushing, obesity, diabetes

## Abstract

**Background:**

Toothbrushing is a health-related lifestyle habit and has been reported to contribute not only to oral health but also to some parameters of general health; however, little research has been conducted to understand the association of the frequency and timing of toothbrushing with the development of comprehensive metabolic abnormalities, with consideration of oral health condition. In this study, using longitudinal data, we examined this association in Japanese adults, adjusting for periodontal condition.

**Methods:**

A 5-year longitudinal study was performed with 4,537 participants between 35 and 64 years old who underwent an annual dental examination in both 2003 and 2008. Data about toothbrushing habits and metabolic abnormalities, such as obesity, hyperglycemia, diabetes, hypertension, hypertriglyceridemia, and low levels of high-density lipoprotein-cholesterol, were analyzed using Poisson regression analysis.

**Results:**

The percentage of participants with a toothbrushing frequency ≤1 time/day was 29.4%, and that for those not brushing their teeth at night was 21.4%. The incidences of obesity and hyperglycemia after 5 years were 5.5% and 28.4%, respectively. A toothbrushing frequency ≤1 time/day was associated with development of obesity (prevalence rate ratio [PRR] 1.77; 95% confidence interval [CI], 1.12–2.80), after adjusting for periodontal condition and potential risk factors. A significant association between not brushing teeth at night and hyperglycemia (PRR 1.30; 95% CI, 1.02–1.66) was observed in participants with toothbrushing frequency of 1 time/day. No association was found between toothbrushing habits and other metabolic abnormalities.

**Conclusions:**

This study suggests that toothbrushing habits are associated with the development of obesity and hyperglycemia.

## INTRODUCTION

In modern affluent societies, unhealthy lifestyle factors are responsible for a large proportion of morbidities and mortalities. For example, smoking, unhealthy dietary habits, lack of regular physical activity, high alcohol consumption, and not maintaining an appropriate body weight have been shown to have an unfavorable influence on cardiovascular disease,^1^^,^^2^ as well as diabetes mellitus^3^ and hypertension.^4^ Additionally, metabolic syndrome, a precursor of cardiovascular disease and diabetes mellitus,^5^ is affected by lifestyle factors, including smoking, diet, physical activity, and alcohol consumption habits.^6^

These lifestyle factors have been shown to be connected to oral health behaviors, such as toothbrushing, as it has been reported that lower toothbrushing frequency correlates with smoking, unhealthier dietary habits, lower physical activity, and higher consumption of alcohol.^7^^,^^8^ Recent studies have shown that low toothbrushing frequency is associated with obesity,^9^ metabolic syndrome,^10^ diabetes mellitus,^11^^,^^12^ and cardiovascular disease.^13^ Furthermore, it has been found that not brushing teeth at night is related to mortality.^14^ These previous studies suggest both the clinical and public health importance of toothbrushing, as toothbrushing leads not only to the prevention of oral disease, but also to a reduced risk of health deterioration. Toothbrushing may be an easily applicable preventive method for overall health deterioration. Additionally, toothbrushing may have the hidden potential to become another factor of improving health deterioration when general lifestyle modification has not been effective.

However, these studies^10^^–^^14^ did not simultaneously address oral health condition. Metabolic abnormalities, including obesity, hyperglycemia, dyslipidemia, and high blood pressure, are associated with poor oral health condition, especially periodontal disease.^15^ The association between toothbrushing and metabolic abnormalities may be influenced not only by lifestyle but also by periodontal condition.^16^ We previously reported that low toothbrushing frequency was associated with the onset of metabolic syndrome, defined by ≥3 components of metabolic syndrome, after adjusting for lifestyle factors and periodontal condition.^17^ The previous study suggests the possibility that toothbrushing is an independent related factor for the development of metabolic syndrome. However, it remained unclear as to which specific metabolic abnormalities were associated with toothbrushing habits and whether this association differed based on the timing of toothbrushing.

In the present study, we examined whether toothbrushing habits (toothbrushing frequency and toothbrushing at night) was longitudinally associated with the development of metabolic abnormalities, including diabetes and hypertension, with consideration of oral health condition in Japanese adults. We hypothesized that toothbrushing was an independent risk factor for developing metabolic abnormalities.

## METHODS

### Study population

This longitudinal study used the medical and dental records of Japanese adults who had undergone workplace health check-ups in 2003 and 2008 at the health examination center of a manufacturing company in Yokohama, Japan. Details of the study design have been described previously.^18^

**Figure 1.**
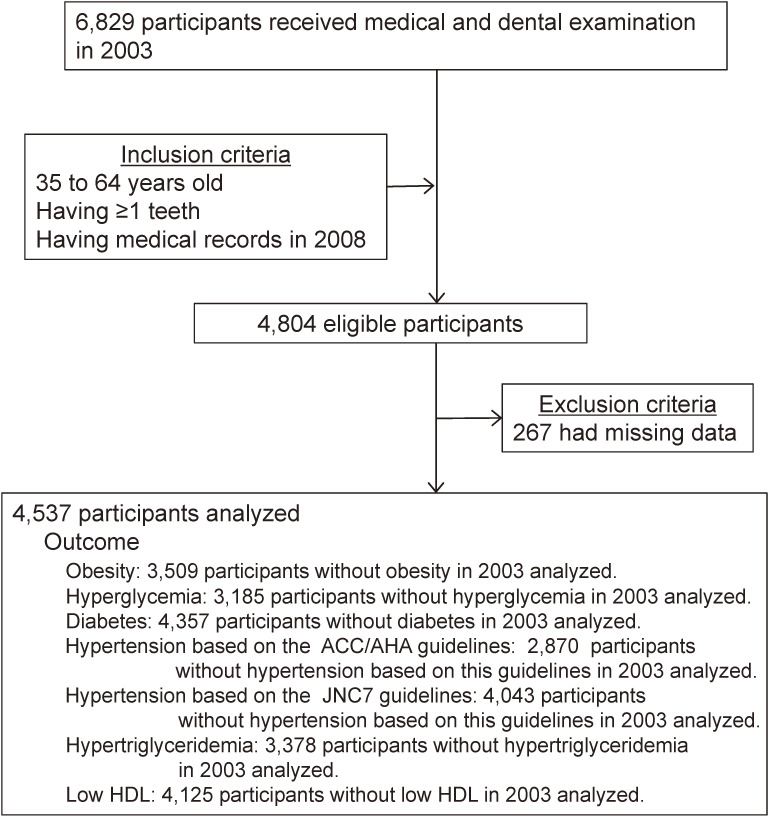
Flow diagram of the study participants. Of the 4,537 overall participants, we selected 3,509 participants when examining the incidence of obesity during a 5-year period, 3,185 participants for hyperglycemia, 4,357 participants for diabetes, 2,870 participants for hypertension based on ACC/AHA guidelines, 4,043 participants for hypertension based on the JNC7 guidelines, 3,378 participants for hypertriglyceridemia, and 4,125 participants for low HDL. ACC/AHA, American College of Cardiology/American Heart Association; HDL, high-density lipoprotein; JNC7, Seventh Report of the Joint National Committee on the Prevention, Detection, Evaluation, and Treatment of High Blood Pressure.

At baseline, 6,829 individuals received both medical and dental examinations. Participants were included if they fulfilled the following criteria at baseline: (1) were 35 to 64 years old, (2) had 1 or more teeth, and (3) had medical records from 2008. Of the 4,804 participants fulfilling the inclusion criteria, 267 participants with missing data were excluded. Finally, 4,537 participants were analyzed in this study (3,643 men and 894 women) (Figure 1).

All participants provided written informed consent for the use of their anonymous data for research. The study design was approved by the Ethics Committee of the Kyushu University Faculty of Dental Sciences, Fukuoka, Japan.

### Assessment of toothbrushing and oral health condition

Toothbrushing habits were recorded based on self-reported questionnaires. The frequency of toothbrushing and the time of day when toothbrushing was performed were derived from the following question: ‘How often do you brush your teeth?’. The response options were: ‘once in the morning’; ‘once in the afternoon’; ‘once at night’; ‘two times, in the morning and at night’; ‘two times, in the morning and the afternoon’; ‘two times, in the afternoon and at night’; ‘three times’; and ‘four and more times’. Then, the ‘once in the morning’, ‘once in the afternoon’, and ‘two times in the morning and afternoon’ were combined to create a dichotomous variable to identify the no toothbrushing at night group. Toothbrushing time was also recorded.

Number of teeth, periodontal condition, and oral hygiene status were recorded as indicators of oral health condition at the baseline examination. A periodontal examination was performed on all teeth except the third molars at two sites (mesiobuccal and mid-buccal), following the National Health and Nutrition Examination Survey III method; probing pocket depth (PPD) and clinical attachment level (CAL) were assessed. The percentage of teeth that bled upon probing (%BOP) was also examined. The periodontal examination was described previously in more detail.^18^ Oral hygiene status was recorded using the Oral Hygiene Index-Simplified.^19^

### Assessment of metabolic abnormalities

The assessment of metabolic abnormalities included the following measurements: fasting glucose, triglyceride, and high-density lipoprotein (HDL) cholesterol concentrations; systolic and diastolic blood pressure; and body mass index (BMI), which was calculated after body weight and height were measured. Metabolic abnormalities were assessed according to the Joint Interim Statement using the criteria for Asians^20^ as follows: obesity was defined as BMI ≥25.0 kg/m^2^ (appropriate cut-off for obesity in Asian individuals^21^); hyperglycemia was defined as fasting glucose ≥100 mg/dL or current antidiabetic treatment; hypertriglyceridemia was defined as triglycerides ≥150 mg/dL or the use of any lipid-lowering medications; and low HDL cholesterol was defined as <40 mg/dL in males and <50 mg/dL in females. Diabetes was determined by fasting glucose ≥126 mg/dL or current antidiabetic treatment.^22^ The oral glucose tolerance test was not conducted, so the diagnostic criteria of diabetes used in this study did not correspond with the current diagnostic criteria proposed by the American Diabetes Association. Hypertension was defined using criteria from the 2017 American College of Cardiology/American Heart Association (ACC/AHA) guidelines and the Seventh Report of the Joint National Committee on the Prevention, Detection, Evaluation, and Treatment of High Blood Pressure (JNC7). In the 2017 ACC/AHA guidelines, hypertension was defined as blood pressure ≥130/80 mm Hg or current antihypertensive treatment,^23^ and hypertension in the JNC7 was defined as blood pressure ≥140/90 mm Hg or current antihypertensive treatment.^24^

### Assessment of other variables

Information about eating habits, smoking, alcohol consumption, physical activity, sleeping hours, and profession was collected using a self-reported questionnaire. Eating habits were assessed using six questions, and three answer categories were collapsed into dichotomous variables as follows: (1) eating a daily snack between meals: yes versus no; (2) preferred taste of food: salty versus normal or lightly salted; (3) skipping breakfast: yes versus no; (4) eating meat and oily food: more versus normal or lower; (5) eating sweet food: more versus normal or lower; and (6) eating dinner: eating home-cooked meals versus eating outside the home or eating take-out food (ie, seldom eat home-cooked meals). Smoking status was categorized as current smoking or nonsmoker. Alcohol consumption was measured via frequency of alcohol consumption and volume of alcoholic beverages consumed; alcohol consumption was categorized into high consumption (≥30 g ethanol per day for men and ≥20 g ethanol per day for women) or no consumption. Physical activity was assessed via frequency of exercise and was grouped as regular (more than 1 day per week) or none. Sleeping hours were divided into ≤5 hours, 6 hours, and ≥7 hours. Occupational status was classified into office workers, skilled workers, and other workers.

### Statistical analysis

Bivariate tests, including the Mann-Whitney *U* test, the Kruskal-Wallis test, and the chi-squared test, were performed to describe oral condition and lifestyle factors according to toothbrushing habits. As logistic regression might have overestimated the risk of association in a high incidence of outcomes,^25^ a Poisson regression model with robust standard errors was used.^26^ The association between toothbrushing habits, including toothbrushing frequency and brushing teeth at night, at baseline (independent variable) and developing each metabolic abnormality (dependent variable) was assessed using Poisson regression analysis. The prevalence rate ratio (PRR) with 95% confidence intervals (CIs) based on robust standard errors was calculated. The Poisson regression models included age, sex, mean CAL, number of teeth, BMI, eating habits (eating snacks between meals, preferring salty dishes, skipping breakfast, eating meat and oily food, eating sweet foods, and seldom eating home-cooked meals), smoking, alcohol consumption, physical activity, sleeping hours, and job as covariates. The model with each metabolic abnormality, except obesity, as the dependent variable additionally included the baseline value of each metabolic abnormality as a covariate (eg, the model for the development of hypertension additionally included systolic blood pressure at baseline). To determine whether brushing teeth at night was associated with metabolic abnormalities regardless of toothbrushing frequency, data stratified by toothbrushing frequency were analyzed. SPSS software (version 25.0 for Windows; IBM SPSS Japan, Tokyo, Japan) was used for data analysis. A two-sided value of *P* < 0.05 was considered statistically significant in all analyses.

## RESULTS

### Characteristics of the participants

Age, obesity, and mean values of CAL were different between the analyzed participants and the participants who did not receive a medical examination after the 5-year follow-up period (Table 1). The toothbrushing habits and metabolic conditions were similar between the participants. The prevalence of each metabolic abnormality at baseline was 22.7% for obesity, 29.7% for hyperglycemia, 3.8% for diabetes, 36.7% for hypertension based on the 2017 ACC/AHA guidelines, 10.9% for hypertension based on the JNC7, 25.5% for hypertriglyceridemia, and 9.1% for low HDL cholesterol (Table 1).

**Table 1.** Descriptive statistics comparing analyzed participants with participants who dropped out of the health examination in 2008

	Analyzed	Dropped out	*P*-value
	(*n* = 4,537)	(*n* = 1,659)	
Age, years, mean (SD)	45.0 (8.4)	46.9 (9.2)	<0.001
Sex			<0.001
Men	3,643 (80.3)	1,203 (72.5)	
Women	894 (19.7)	456 (27.5)	
Obesity			0.015
BMI <25.0 kg/m^2^	3,509 (77.3)	1,234 (74.4)	
BMI ≥25.0 kg/m^2^	1,028 (22.7)	425 (25.6)	
Fasting glucose^a^			0.518
Normal	3,185 (70.2)	1,150 (69.3)	
Elevated	1,346 (29.7)	506 (30.5)	
Diabetes^a^			0.102
No	4,357 (96.2)	1,577 (95.1)	
Yes	174 (3.8)	79 (4.8)	
Hypertension based on the 2017 ACC/AHA guidelines			0.911
No	2,870 (63.3)	1,052 (63.4)	
Yes	1,667 (36.7)	607 (36.6)	
Hypertension based on the JNC7 guidelines			0.070
No	4,043 (89.1)	1,451 (87.5)	
Yes	494 (10.9)	208 (12.5)	
Triglycerides			0.743
Normal	3,378 (74.5)	1,242 (74.9)	
Elevated	1,159 (25.5)	417 (25.1)	
HDL cholesterol			0.593
Normal	4,125 (90.9)	1,501 (90.5)	
Reduced	412 (9.1)	158 (9.5)	

Toothbrushing frequency			0.600
≥3 times	844 (18.6)	304 (18.3)	
2 times	2,361 (52.0)	846 (51.0)	
≤1 time	1,332 (29.4)	509 (30.7)	
Toothbrushing at night			0.983
Yes	3,565 (78.6)	1,304 (78.6)	
No	972 (21.4)	355 (21.4)	
CAL, mean (SD)	2.52 (0.72)	2.57 (0.77)	0.018

ACC/AHA, American College of Cardiology/American Heart Association; BMI, body mass index; CAL, clinical attachment level; HDL, high-density lipoprotein; JNC7, Seventh Report of the Joint National Committee on the Prevention, Detection, Evaluation, and Treatment of High Blood Pressure; SD, standard deviation.

Values reported as *n* (%), unless otherwise noted.

Chi-square test was performed for categorical variables, and Mann-Whitney *U* test was performed for continuous variables.

^a^Excluding individuals with a missing value (*n* = 6 in analyzed and *n* = 3 in dropped out).

### Toothbrushing habits, oral condition, and lifestyle factors

The percentage of participants with a toothbrushing frequency of ≤1 time/day and who did not brushing their teeth at night was 29.4% and 21.4%, respectively. The association between toothbrushing frequency and brushing teeth at night is shown in Table 2. Of the 1,332 participants with a toothbrushing frequency of 1 time/day, 885 brushed their teeth in the morning, 10 in the afternoon, and 437 at night. Participants with a toothbrushing frequency of ≤1 time/day had higher mean values of CAL, more plaque and calculus, higher %BOP, a greater prevalence of current smoking, higher alcohol consumption, lower physical activity, greater preference for salty foods, greater prevalence of skipping breakfast, higher consumption of meat and oily food, lower consumption of home-cooked meals and shorter sleeping hours than those with a toothbrushing frequency of ≥3 times/day (Table 3). The association of not brushing teeth at night with oral condition and lifestyle factors was similar to that of toothbrushing frequency.

**Table 2.** Association between toothbrushing frequency and toothbrushing at night

	Toothbrushing frequency			
	≤1 time	2 times	≥3 times	*P*-value
	(*n* = 1,332)	(*n* = 2,361)	(*n* = 844)	
Toothbrush at night				<0.001
Yes	437 (32.8)	2,284 (96.7)	844 (100)	
No	895 (67.2)	77 (3.3)	0 (0)	

*n* (%), Chi-square test.

**Table 3.** Oral condition and lifestyle factors according to toothbrushing habits at baseline

	Toothbrushing frequency				Brushing teeth at night		
	≤1 time	2 times	≥3 times	*P*-value	No	Yes	*P*-value
	(*n* = 1,332)	(*n* = 2,361)	(*n* = 844)		(*n* = 972)	(*n* = 3,565)	
Age, years, mean (SD)	44.9 (8.3)	44.9 (8.3)	45.8 (8.5)	0.020	44.5 (8.2)	45.2 (8.4)	0.022
Men, %	94.5	80.6	57.1	<0.001	76.1	95.7	<0.001
CAL, mean (SD)	2.55 (0.72)	2.52 (0.73)	2.46 (0.67)	0.015	2.57 (0.73)	2.50 (0.71)	0.005
Dental plaque (debris index simplified), mean (SD)	0.81 (0.44)	0.69 (0.39)	0.58 (0.38)	<0.001	0.80 (0.45)	0.68 (0.40)	<0.001
Calculus (calculus index simplified), mean (SD)	0.57 (0.51)	0.45 (0.44)	0.36 (0.39)	<0.001	0.58 (0.52)	0.44 (0.43)	<0.001
%BOP, mean (SD)	24.1 (23.0)	19.4 (21.0)	17.1 (19.6)	<0.001	23.7 (22.2)	19.5 (21.2)	<0.001
Number of teeth, mean (SD)	28.0 (2.5)	27.8 (2.6)	27.7 (2.4)	0.049	28.0 (2.5)	27.8 (2.6)	0.009
Occupational status, %				<0.001			<0.001
Office workers	29.8	27.6	40.5		30.1	30.8	
Skilled workers	40.5	35.2	21.1		41.4	32.2	
Others	29.7	37.1	38.4		28.5	37.0	
Current smoking, %	38.4	24.9	11.8	<0.001	40.2	22.7	<0.001
High alcohol consumption, %	30.0	25.2	17.3	<0.001	34.1	22.7	<0.001
Lack of regular physical activity, %	70.0	64.3	53.9	<0.001	70.2	62.3	<0.001
Eats snacks between meals, %	27.1	31.5	42.1	<0.001	24.7	34.2	<0.001
Prefers salty dishes, %	17.5	13.0	9.5	<0.001	20.4	11.9	<0.001
Skips breakfast, %	26.6	12.9	7.7	<0.001	28.0	12.7	<0.001
Eats more meat and oily food, %	21.1	15.8	10.9	<0.001	23.8	14.4	<0.001
Eats more sweet food, %	11.8	13.0	13.5	<0.001	10.7	13.3	0.033
Seldom eats home-cooked meals for dinner, %	27.0	19.6	13.4	<0.001	28.0	18.6	<0.001
Sleeping hours, %				0.028			0.004
≥7 hours	32.7	33.9	36.7		32.0	34.6	
6 hours	44.7	46.3	46.1		44.1	46.3	
≤5 hours	22.6	19.8	17.2		23.9	19.1	

CAL, clinical attachment level; %BOP, percentage of sites that bled upon probing; SD, standard deviation.

Chi-square test was used for categorical variables, and Mann-Whitney *U* test or Kruskal-Wallis test was used for continuous variables.

### Toothbrushing habits and development of metabolic abnormalities

After excluding participants with each metabolic abnormality at baseline, the incidence of the development of each metabolic abnormality was calculated. We analyzed 3,509 participants when examining incidences of obesity, 3,185 for hyperglycemia, 3,378 for hypertriglyceridemia, 4,357 for diabetes, 2,870 for hypertension based on the 2017 ACC/AHA guidelines, 4,043 for hypertension based on the JNC7 guidelines, 3,378 for hypertriglyceridemia, and 4,125 for low HDL cholesterol (Table 4). The incidence of the development of each metabolic abnormality was 5.5% for obesity, 28.4% for hyperglycemia, 2.3% for diabetes, 16.8% for hypertension based on the 2017 ACC/AHA guidelines, 7.9% for hypertension based on the JNC7, 12.8% for hypertriglyceridemia, and 2.7% for low HDL cholesterol (Table 4).

**Table 4.** Incidence of each metabolic abnormality

	Without each metabolic abnormality at baseline	Incidence of each metabolic abnormality
Obesity	3,509	5.5%
Hyperglycemia	3,185	28.4%
Diabetes	4,357	2.3%
Hypertension based on the 2017 ACC/AHA guidelines	2,870	16.8%
Hypertension based on the JNC7 guidelines	4,043	7.9%
Hypertriglyceridemia	3,378	12.8%
Low HDL	4,125	2.7%

ACC/AHA, American College of Cardiology/American Heart Association; JNC7, Seventh Report of the Joint National Committee on the Prevention, Detection, Evaluation, and Treatment of High Blood Pressure.

Poisson regression models showed that a toothbrushing frequency of ≤1 time/day was positively associated with the development of obesity (PRR 1.77; 95% CI, 1.12–2.80) after adjusting for age, sex, number of teeth, mean CAL, BMI, eating snacks between meals, preferring salty dishes, skipping breakfast, eating meat and oily food, eating sweet food, seldom eating home-cooked meals, smoking, consuming alcohol, physical activity, sleeping hours, job, and the baseline value of each metabolic abnormality (Table 5). Not brushing teeth at night was significantly associated with the development of obesity (PRR 1.49; 95% CI, 1.13–1.96) and hyperglycemia (PRR 1.14; 95% CI, 1.00–1.29) (Table 6). When we examined the association between brushing teeth at night and each metabolic abnormality stratified by toothbrushing frequency, participants who had a toothbrushing frequency of 1 time/day and were not brushing at night were more likely to develop hyperglycemia (PRR 1.30; 95% CI, 1.02–1.66) than those brushing at night (Table 7). A significant association between brushing at night and obesity was not found (Table 7). Regarding periodontal condition, mean CAL, PPD, calculus, and %BOP were higher in participants who had a toothbrushing frequency of 1 time/day and were not brushing at night than in those who brushed at night (eTable 1). In participants with toothbrushing frequency of 1 time/day, not brushing at night was not significantly associated with hyperglycemia in the model with %BOP (eTable 2).

**Table 5.** Association between toothbrushing frequency and the development of each metabolic abnormality

	Development of each metabolic abnormality		Crude PRR (95% CI)	Adjusted PRR^a^ (95% CI)	
	No	Yes		Model 1	Model 2
Obesity					
Toothbrushing frequency, *n* (%)					
≥3 times	692 (97.2)	20 (2.8)	1	1	1
2 times	1,757 (94.8)	96 (5.2)	1.84 (1.15–2.96)	1.22 (0.78–1.89)	1.20 (0.77–1.88)
≤1 time	866 (91.7)	78 (8.3)	2.94 (1.82–4.76)	1.80 (1.14–2.84)	1.77 (1.12–2.80)
CAL, mean (SD)	2.49 (0.72)	2.58 (0.74)	1.16 (0.99–1.35)		1.13 (0.97–1.31)
Hyperglycemia					
Toothbrushing frequency, *n* (%)					
≥3 times	495 (77.8)	141 (22.2)	1	1	1
2 times	1,224 (72.0)	475 (28.0)	1.26 (1.07–1.49)	1.03 (0.88–1.21)	1.04 (0.89–1.21)
≤1 time	561 (66.0)	289 (34.0)	1.53 (1.29–1.82)	1.14 (0.96–1.38)	1.14 (0.96–1.36)
CAL, mean (SD)	2.47 (0.67)	2.51 (0.72)	1.05 (0.97–1.14)		0.98 (0.91–1.06)
Diabetes					
Toothbrushing frequency, *n* (%)					
≥3 times	808 (98.7)	11 (1.3)	1	1	1
2 times	2,234 (98.2)	42 (1.8)	1.37 (0.71–2.66)	1.02 (0.56–1.85)	1.02 (0.56–1.87)
≤1 time	1,215 (96.3)	47 (3.7)	2.77 (1.45–5.31)	1.62 (0.91–2.88)	1.62 (0.91–2.88)
CAL, mean (SD)	2.51 (0.70)	2.71 (1.12)	1.35 (1.11–1.65)		1.07 (0.78–1.47)
Hypertension based on the 2017 ACC/AHA guidelines					
Toothbrushing frequency, *n* (%)					
≥3 times	502 (87.8)	70 (12.2)	1	1	1
2 times	1,271 (83.5)	251 (16.5)	1.35 (1.05–1.72)	1.14 (0.90–1.45)	1.14 (0.90–1.45)
≤1 time	616 (79.4)	160 (20.6)	1.69 (1.30–2.18)	1.27 (0.97–1.66)	1.27 (0.97–1.66)
CAL, mean (SD)	2.47 (0.70)	2.55 (0.75)	1.13 (1.02–1.25)		1.03 (0.93–1.15)
Hypertension based on the JNC7 guidelines					
Toothbrushing frequency, *n* (%)					
≥3 times	710 (92.6)	57 (7.4)	1	1	1
2 times	1,955 (92.3)	163 (7.7)	1.04 (0.78–1.38)	0.92 (0.70–1.23)	0.93 (0.70–1.23)
≤1 time	1,057 (91.3)	101 (8.7)	1.17 (0.86–1.60)	0.88 (0.64–1.22)	0.89 (0.64–1.22)
CAL, mean (SD)	2.50 (0.71)	2.62 (0.80)	1.21 (1.07–1.36)		1.09 (0.96–1.25)
Hypertriglyceridemia					
Toothbrushing frequency, *n* (%)					
≥3 times	644 (91.5)	60 (8.5)	1	1	1
2 times	1,563 (87.2)	230 (12.8)	1.51 (1.15–1.97)	1.12 (0.87–1.45)	1.12 (0.87–1.45)
≤1 time	738 (83.8)	143 (16.2)	1.90 (1.43–2.53)	1.24 (0.94–1.63)	1.23 (0.94–1.63)
CAL, mean (SD)	2.50 (0.73)	2.51 (0.68)	1.01 (0.90–1.14)		1.04 (0.93–1.17)
Low HDL					
Toothbrushing frequency, *n* (%)					
≥3 times	786 (97.6)	19 (2.4)	1	1	1
2 times	2,102 (97.4)	56 (2.6)	1.07 (0.64–1.80)	1.06 (0.65–1.73)	1.08 (0.66–1.76)
≤1 time	1,143 (96.9)	37 (3.1)	1.30 (0.75–2.24)	1.21 (0.67–2.20)	1.23 (0.68–2.23)
CAL, mean (SD)	2.51 (0.72)	2.44 (0.62)	0.88 (0.68–1.13)		0.86 (0.67–1.11)

ACC/AHA, American College of Cardiology/American Heart Association; CI, confidence interval; CAL, clinical attachment level; HDL, high-density lipoprotein; JNC7, Seventh Report of the Joint National Committee on the Prevention, Detection, Evaluation, and Treatment of High Blood Pressure; PRR, prevalence rate ratio.

Poisson regression models with robust standard error; each metabolic abnormality was the dependent variable and toothbrushing frequency was the independent variable.

The crude model included one independent variable and one dependent variable.

^a^Model 1 adjusted for age, sex, number of teeth, BMI, eating snacks between meals, preferring salty dishes, skipping breakfast, eating meat and oily food, eating sweet food, seldom eating home-cooked meals, smoking, alcohol consumption, physical activity, sleeping hours, job, and baseline value of each metabolic abnormality, and model 2 additionally included mean CAL.

**Table 6.** Association between brushing teeth at night and the development of each metabolic abnormality

	Development of each metabolic abnormality		Crude PRR (95% CI)	Adjusted PRR^a^ (95% CI)	
	No	Yes		Model 1	Model 2
Obesity					
Brushing teeth at night, *n* (%)					
Yes	2,702 (95.2)	135 (4.8)	1	1	1
No	613 (91.2)	59 (8.8)	1.85 (1.37–2.48)	1.51 (1.14–1.99)	1.49 (1.13–1.96)
CAL, mean (SD)	2.49 (0.72)	2.58 (0.74)	1.16 (0.99–1.35)		1.13 (0.97–1.32)
Hyperglycemia					
Brushing teeth at night, *n* (%)					
Yes	1,898 (73.5)	685 (26.5)	1	1	1
No	382 (63.5)	220 (36.5)	1.38 (1.22–1.56)	1.14 (1.00–1.29)	1.14 (1.00–1.29)
CAL, mean (SD)	2.47 (0.67)	2.51 (0.72)	1.05 (0.97–1.14)		0.98 (0.91–1.06)
Diabetes					
Brushing teeth at night, *n* (%)					
Yes	3,369 (98.0)	68 (2.0)	1	1	1
No	888 (96.5)	32 (3.5)	1.76 (1.16–2.66)	1.08 (0.73–1.60)	1.07 (0.72–1.60)
CAL, mean (SD)	2.51 (0.70)	2.71 (1.12)	1.35 (1.11–1.65)		1.09 (0.80–1.48)
Hypertension based on the 2017 ACC/AHA guidelines					
Brushing teeth at night, *n* (%)					
Yes	1,960 (84.4)	361 (15.6)	1	1	1
No	429 (78.1)	120 (21.9)	1.41 (1.17–1.69)	1.16 (0.97–1.40)	1.16 (0.96–1.39)
CAL, mean (SD)	2.47 (0.70)	2.55 (0.75)	1.13 (1.02–1.25)		1.03 (0.92–1.14)
Hypertension based on the JNC7 guidelines					
Brushing teeth at night, *n* (%)					
Yes	2,955 (92.4)	243 (7.6)	1	1	1
No	767 (90.8)	78 (9.2)	1.21 (0.95–1.55)	1.02 (0.80–1.31)	1.02 (0.80–1.31)
CAL, mean (SD)	2.50 (0.71)	2.62 (0.80)	1.21 (1.07–1.36)		1.09 (0.96–1.25)
Hypertriglyceridemia					
Brushing teeth at night, *n* (%)					
Yes	2,424 (88.2)	324 (11.8)	1	1	1
No	521 (82.7)	109 (17.3)	1.47 (1.20–1.79)	1.08 (0.89–1.31)	1.08 (0.89–1.31)
CAL, mean (SD)	2.50 (0.73)	2.51 (0.68)	1.01 (0.90–1.14)		1.04 (0.93–1.17)
Low HDL					
Brushing teeth at night, *n* (%)					
Yes	3,174 (97.3)	89 (2.7)	1	1	1
No	839 (97.3)	23 (2.7)	0.98 (0.62–1.54)	0.93 (0.58–1.49)	0.94 (0.59–1.50)
CAL, mean (SD)	2.51 (0.72)	2.44 (0.62)	0.88 (0.68–1.13)		0.87 (0.67–1.12)

ACC/AHA, American College of Cardiology/American Heart Association; CI, confidence interval; CAL, clinical attachment level; HDL, high-density lipoprotein; JNC7, Seventh Report of the Joint National Committee on the Prevention, Detection, Evaluation, and Treatment of High Blood Pressure; PRR, prevalence rate ratio.

Poisson regression models with robust standard error; each metabolic abnormality was the dependent variable and not brushing teeth at night was the independent variable.

The crude model included one independent variable and one dependent variable.

^a^Model 1 adjusted for age, sex, number of teeth, BMI, eating snacks between meals, preferring salty dishes, skipping breakfast, eating meat and oily food, eating sweet food, seldom eating home-cooked meals, smoking, alcohol consumption, physical activity, sleeping hours, job, and baseline value of each metabolic abnormality, and model 2 additionally included mean CAL.

**Table 7.** Association between toothbrushing at night and the development of each metabolic abnormality based on toothbrushing frequency

	Development of each metabolic abnormality		Crude PRR (95% CI)	Adjusted PRR (95% CI)^a^	
	No	Yes		Model 1	Model 2
*Twice a day toothbrushing*					
Obesity					
Toothbrushing at night					
Yes	1,699 (94.9)	91 (5.1)	1	1	1
No	58 (92.1)	5 (7.9)	1.56 (0.66–3.71)	1.48 (0.66–3.32)	1.49 (0.66–3.33)
CAL, mean (SD)	2.50 (0.74)	2.52 (0.71)	1.05 (0.83–1.32)		1.03 (0.80–1.32)
Hyperglycemia					
Toothbrushing at night					
Yes	1,190 (71.9)	465 (28.1)	1	1	1
No	34 (77.3)	10 (22.7)	0.81 (0.47–1.40)	0.68 (0.39–1.19)	0.68 (0.39–1.19)
CAL, mean (SD)	2.47 (0.68)	2.53 (0.78)	0.99 (0.87–1.15)		1.00 (0.90–1.11)
Diabetes					
Toothbrushing at night					
Yes	2,163 (98.2)	40 (1.8)	1	1	1
No	71 (97.3)	2 (2.7)	1.51 (0.37–6.12)	0.77 (0.19–3.09)	0.79 (0.19–3.28)
CAL, mean (SD)	2.51 (0.71)	2.79 (1.47)	1.39 (1.08–1.79)		1.38 (1.04–1.83)
Hypertension in 2017 ACC/AHA guideline					
Toothbrushing at night					
Yes	1,228 (83.6)	241 (16.4)	1	1	1
No	43 (81.1)	10 (18.9)	1.15 (0.65–2.03)	1.11 (0.66–1.90)	1.11 (0.65–1.89)
CAL, mean (SD)	2.48 (0.73)	2.57 (0.74)	1.14 (1.00–1.29)		1.07 (0.93–1.24)
Hypertension in JNC7					
Toothbrushing at night					
Yes	1,890 (92.3)	158 (7.7)	1	1	1
No	65 (92.9)	5 (7.1)	0.93 (0.39–2.18)	1.09 (0.50–2.38)	1.10 (0.50–2.41)
CAL, mean (SD)	2.50 (0.74)	2.58 (0.73)	1.13 (0.97–1.33)		1.03 (0.86–1.24)
Hypertriglyceridemia					
Toothbrushing at night					
Yes	1,516 (87.2)	222 (12.8)	1	1	1
No	47 (85.5)	8 (14.5)	1.14 (0.59–2.19)	0.85 (0.44–1.61)	0.84 (0.44–1.60)
CAL, mean (SD)	2.51 (0.75)	2.49 (0.68)	0.98 (0.84–1.14)		1.04 (0.89–1.21)
Low HDL					
Toothbrushing at night					
Yes	2,031 (97.4)	55 (2.6)	1		
No	71 (98.6)	1 (1.4)	0.53 (0.07–3.75)		
CAL, mean (SD)	2.51 (0.73)	2.57 (0.71)	0.70 (0.45–1.09)		

*Once a day toothbrushing*					
Obesity					
Toothbrushing at night					
Yes	238 (94.4)	14 (5.6)	1	1	1
No	402 (91.4)	38 (8.6)	1.56 (0.86–2.81)	1.20 (0.66–2.17)	1.15 (0.63–2.12)
CAL, mean (SD)	2.52 (0.71)	2.73 (0.74)	1.39 (1.05–1.84)		1.12 (0.85–2.12)
Hyperglycemia					
Toothbrushing at night					
Yes	156 (73.9)	55 (26.1)	1	1	1
No	245 (61.1)	156 (38.9)	1.49 (1.15–1.93)	1.30 (1.02–1.66)	1.30 (1.02–1.66)
CAL, mean (SD)	2.51 (0.67)	2.55 (0.66)	1.05 (0.90–1.23)		0.99 (0.84–1.16)
Diabetes					
Toothbrushing at night					
Yes	280 (96.6)	10 (3.4)	1	1	1
No	587 (96.2)	23 (3.8)	1.09 (0.53–2.27)	0.56 (0.24–1.31)	0.57 (0.24–1.36)
CAL, mean (SD)	2.55 (0.70)	2.76 (0.85)	1.42 (0.95–2.12)		0.94 (0.63–1.39)
Hypertension in 2017 ACC/AHA guideline					
Toothbrushing at night					
Yes	158 (82.3)	34 (17.7)	1	1	1
No	268 (77.0)	80 (23.0)	1.30 (0.91–1.86)	1.17 (0.81–1.67)	1.17 (0.81–1.69)
CAL, mean (SD)	2.51 (0.67)	2.56 (0.79)	1.09 (0.86–1.39)		0.98 (0.77–1.24)
Hypertension in JNC7					
Toothbrushing at night					
Yes	245 (92.5)	20 (7.5)	1	1	1
No	504 (90.5)	53 (9.5)	1.26 (0.77–2.06)	1.10 (0.67–1.79)	1.09 (0.66–1.77)
CAL, mean (SD)	2.54 (0.72)	2.67 (0.79)	1.24 (0.95–1.60)		1.11 (0.86–1.44)
Hypertriglyceridemia					
Toothbrushing at night					
Yes	193 (86.9)	29 (13.1)	1	1	1
No	356 (84.0)	68 (16.0)	1.23 (0.82–1.84)	0.87 (0.57–1.33)	0.87 (0.57–1.32)
CAL, mean (SD)	2.52 (0.70)	2.60 (0.74)	1.15 (0.90–1.46)		0.96 (0.90–1.02)
Low HDL					
Toothbrushing at night					
Yes	266 (96.7)	9 (3.3)	1	1	1
No	549 (97.2)	16 (2.8)	0.87 (0.39–1.93)	1.14 (0.48–2.73)	1.23 (0.52–2.89)
CAL, mean (SD)	2.54 (0.70)	2.40 (0.57)	0.73 (0.41–1.28)		0.71 (0.38–1.34)

CI, confidence interval; CAL, clinical attachment level; HDL, high-density lipoprotein; PRR, prevalence rate ratio.

Poisson regression models with robust standard error; each metabolic abnormalities was the dependent variable and no toothbrushing at night was the independent variable.

The crude model included one independent variable and dependent variable.

^a^Model 1 adjusted for age, sex, number of teeth, BMI, eating snacks between meals, preferring salty dishes, skipping breakfast, eating meat and oily food, eating sweet food, seldom eating home-cooked meals, smoking, alcohol consumption, physical activity, sleeping hours, job, and baseline value of each metabolic abnormality, and model 2 additionally included mean CAL.

## DISCUSSION

We found that a low frequency of toothbrushing was associated with the development of obesity after adjusting for covariates and, furthermore, that not brushing teeth at night in participants with toothbrushing frequency of 1 time/day was associated with hyperglycemia. This longitudinal study reinforced the results of a previous cross-sectional study,^9^ which showed the association between a low frequency of toothbrushing and obesity. Moreover, the current study extends the findings of prior longitudinal studies^10^^,^^12^^,^^17^ in regard to toothbrushing frequency and health conditions by specifying the link between the time of day of toothbrushing and metabolic abnormalities.

The association between toothbrushing habits and obesity was found, even though periodontal condition and several lifestyle factors were adjusted for in multivariate models, suggesting that this association is explained by other related factors. Our results are similar to those of a previous study demonstrating a cross-sectional association between low toothbrushing frequency and obesity after adjusting for lifestyle factors and periodontal condition (odds ratio [OR] 1.22 of 1 time/day toothbrushing and OR 1.48 of 0 times/day toothbrushing).^9^ In this study, PRR for obesity with ≤1 time/day toothbrushing was 1.77, indicating a relatively stronger association than that in the Park et al study.^9^ This might be related to unobserved confounders, such as diet quality, including total energy intake and vegetable and fruit intake.

One possible pathway leading from toothbrushing to obesity may involve endotoxemia. It has been demonstrated that metabolic endotoxemia induced by lipopolysaccharides results in an increase in total body fat mass in mice.^27^ The microbiome of subgingival plaque mainly consists of Gram-negative bacteria that contain a lipopolysaccharide on the outer membrane^28^ and subgingival plaque contributes significantly to endotoxemia. From this perspective, it seems likely that the accumulation of subgingival plaque caused by poor toothbrushing habits contributes to endotoxemia and subsequently leads to obesity. Another possible explanation for the association between toothbrushing and obesity may be related to leptin, which regulates appetite and energy balance primarily through hypothalamic neurons in the central nervous system. Oral proprioceptive signals modulate the hypothalamic histamine neurons, which are concordant with the leptin signaling system.^29^ One study reported that the serum leptin level within 3 hours after a meal was changed after toothbrushing in non-obese young men.^30^ This suggests that mechanical stimulation of the gingiva by toothbrushing may play a role in appetite regulation by influencing leptin, which can control obesity.

The effect of brushing teeth at night on hyperglycemia was observed in participants with toothbrushing frequency of 1 time/day. Not brushing teeth at night in individuals with low frequency of toothbrushing can easily cause poor oral hygiene status, because saliva flow is low while sleeping, and bacterial clearance is reduced, resulting in greater bacterial colonization of oral tissues.^31^ In fact, participants who did not brush their teeth at night had more calculus and higher level of BOP than those who brushed their teeth at night among participants with toothbrushing frequency of 1 time/day (eTable 1). When we added oral hygiene status to the model, the association between not brushing teeth at night and hyperglycemia was attenuated (PRR 1.29; 95% CI, 0.99–1.68 in the model including %BOP; eTable 2), suggesting that gingival inflammation may play a role in mediating this association. However, a significant association between toothbrushing and hyperglycemia remained even in the model adjusted for mean CAL (Table 6 and Table 7). CAL reflects a historical accumulation of periodontal destruction, while BOP is a better real-time indicator of inflammation than CAL.^32^ The present study indicates that the intensity of existing gingival inflammation is a mediator between toothbrushing habits and hyperglycemia. It is considered that gingival inflammation caused by poor toothbrushing is linked with increased systemic inflammation/endothelial dysfunction and low adiponectin levels, which in turn may lead to an increased risk of hyperglycemia.^33^ For individuals who had toothbrushing frequency of once a day without brushing teeth at night, increased frequency of toothbrushing may have the hidden potential to reduce a risk of hyperglycemia. In this context, this study showed a lack of a significant association between toothbrushing habits and diabetes, which was probably due to the low incidence of diabetes (2.3%). A longer follow-up study is necessary to confirm this association.

Although we were not able to clearly explain the direct link between toothbrushing and metabolic abnormalities, one potential pathway may be that the removal of bacterial plaque by toothbrushing decreases chronic low-grade systemic inflammation that can contribute to metabolic abnormalities, such as hyperglycemia. An animal experiment showed that subgingival bacteria, such as *Porphyromonas gingivalis*, induced a change in the bacterial composition of gut microbiota accompanied by increased systemic inflammation,^34^ implying that oral pathogens may lead to metabolic abnormalities.

Our results were different from those of Kuwabara et al,^12^ who found that individuals who did not brush their teeth after every meal had a higher risk of developing diabetes during a 5-year follow-up period than those who brushed their teeth after every meal. However, the study by Kuwabara et al^12^ did not consider lifestyle factors, such as eating habits, smoking, alcohol consumption, physical activity, and sleeping hours, which contribute to diabetes development. Therefore, their results did not exclude the possibility of residual confounding due to unmeasured factors. Our results regarding hyperglycemia are similar to those reported by Kobayashi et al.^10^ Their finding of no association between toothbrushing frequency and hyperglycemia was the same as in this study, although Kobayashi et al did not evaluate the timing of toothbrushing.^10^ More longitudinal studies are required to confirm whether the timing of toothbrushing affects hyperglycemia.

Low frequency of toothbrushing and not brushing teeth at night was inversely associated with eating habits, such as eating snacks between meals and eating more sweet foods (Table 2). This finding may be partly because participants are prone to brushing their teeth after eating a snack or a sweet food to prevent dental caries and maintain oral hygiene. Regarding metabolic abnormalities, participants who ate snacks between meals were less likely to have hyperglycemia, hypertriglyceridemia, and hypertension based on the cross-sectional data (data not shown). It has been reported that individuals who eat snacks more frequently eat more fruits, vegetables, milk, and dairy products and less fried food than those with a lower eating frequency.^35^ The inverse association between eating snacks and metabolic abnormalities may be explained via diet quality.

This study had several limitations. First, diet quality, such as total energy intake and vegetable and fruit intake, was not investigated, although eating habits were included in our study. It has been reported that toothbrushing frequency is positively associated with intake of vegetables and fruits,^36^ which contributes to the prevention of metabolic abnormalities.^37^ We also did not investigate inflammatory parameters, such as high-sensitivity C-reactive protein, which is considered to be responsible for the link between gingival and systemic inflammation. These unmeasured potential confounders may have biased our estimates of the association between toothbrushing habits and metabolic abnormalities owing to using the data of health check-ups; these variables that might have been important were not recorded and could not be included in the model. Future studies are needed to assess these factors. Second, we did not obtain glucose tolerance test data, which defines diabetes more accurately, since a glucose tolerance test is more time consuming in the health examination setting and a fasting blood examination was more practical. Third, we used a partial mouth assessment for periodontal condition, which did not include an examination of lingual or palatal sites. Our results potentially underestimate periodontal condition. Finally, the sample investigated was not representative of the general population because this study included individuals who received workplace health checkups, and the prevalences of obesity and periodontal conditions were different between the analyzed participants and those who dropped out of the follow-up examination. This limitation should be taken into consideration when applying the findings to other populations.

In conclusion, this longitudinal study confirmed that low frequency of toothbrushing was associated with the development of obesity, even after adjusting for potential confounders. An association between not brushing teeth at night and hyperglycemia was found in individuals with a toothbrushing frequency of 1 time/day.

## APPENDIX A. SUPPLEMENTARY DATA

eTable 1. Oral condition according to toothbrushing frequency and brushing teeth at night

eTable 2. Association between toothbrushing at night and hyperglycemia adjusted for mean PPD, oral hygiene status, or %BOP in participants with toothbrushing frequency of once a day and without hyperglycemia at baseline
