# Supplementary material for: Longitudinal Associations of Toothbrushing With Obesity and Hyperglycemia
Source: J Epidemiol. 2020 Dec 5;30(12):556–65. doi: 10.2188/jea.JE20190165 (PMC7661336; doi:10.2188/jea.JE20190165)
Supplement: Supplementary file 1 [file je-30-556-s001.pdf]

eTable 1. Oral condition according to toothbrushing frequency and brushing teeth at night

	Brushing teeth at night		
	No	Yes	p-value[a]
Twice a day toothbrushing			
CAL	2.52 (0.69)	2.52 (0.73)	0.847
PPD	2.16 (0.45)	2.19 (0.47)	0.744
Dental plaque (debris index simplified)	0.65 (0.39)	0.69 (0.39)	0.419
Calculus (calculus index simplified)	0.42 (0.38)	0.45 (0.44)	0.763
%BOP	18.7 (19.6)	19.4 (20.8)	0.884
Number of teeth	27.9 (2.0)	27.8 (2.6)	0.930
Once a day toothbrushing			
CAL	2.61 (0.73)	2.44 (0.65)	<0.001
PPD	2.32 (0.52)	2.16 (0.43)	<0.001
Dental plaque	0.79 (0.44)	0.76 (0.43)	0.239
Calculus	0.58 (0.53)	0.47 (0.45)	0.003
%BOP	23.9 (22.5)	20.4 (21.9)	0.005
Number of teeth	28.0 (2.5)	28.0 (2.2)	0.660

CAL, clinical attachment level; PPD, periodontal pocket depth; %BOP, percentage of sites that bled upon probing; SD, standard deviation.
Values reported as mean (SD)
[a]Mann-Whitney *U* test

eTable 2. Association between toothbrushing at night and hyperglycemia adjusted for mean PPD, oral hygiene status, or %BOP in participants with toothbrushing frequency of once a day and without hyperglycemia at baseline

	Development of hyperglycemia		Crude PRR (95% CI)	Adjusted PRR[a] (95% CI)		
	No	Yes		Model 1	Model 2	Model 3
Once a day toothbrushing						
Toothbrushing at night, n (%)						
Yes	156 (73.9)	55 (26.1)	1	1	1	1
No	245 (61.1)	156 (38.9)	1.49 (1.15–1.93)	1.30 (1.01–1.66)	1.29 (0.99–1.68)	1.29 (1.00–1.66)
PPD, mean (SD)	2.23 (0.45)	2.26 (0.47)	1.10 (0.88–1.38)	0.99 (0.79–1.27)		
%BOP, mean (SD)	22.3 (21.9)	22.7 (21.5)	1.00 (0.99–1.01)		1.00 (0.99–1.00)	
Dental plaque (DIS)	0.78 (0.44)	0.80 (0.42)	1.06 (0.83–1.35)			0.92 (0.68–1.23)
Calculus (CIS)	0.51 (0.49)	0.56 (0.51)	1.12 (0.91–1.38)			1.08 (0.83–1.39)

CI, confidence interval; CIS, calculus index-simplified; DIS, debris index-simplified; HDL, high-density lipoprotein; %BOP, percentage of teeth in bleeding on probing; PPD, probing pocket depth; PRR, prevalence rate ratio.

Poisson regression models with robust standard error; each metabolic abnormalities was the dependent variable and no toothbrushing at night was the independent variable.

The crude model included one independent variable and dependent variable.

Model 1, 2 and 3 included PPD, %BOP, and DIS and CIS, respectively.

[a] Adjusted for age, sex, number of teeth, BMI, taking a snack daily, preferred salty dishes, skipping breakfast, eating meat and oily food, eating sweet food, seldom eating home-cooked meals, smoking, alcohol consumption, physical activity, sleeping hours, job, and baseline value of each metabolic abnormality.
